# Characterization of secondary‐radiation background in X‐ray flat‐panel detectors during scanning proton beam irradiation

**DOI:** 10.1002/mp.70121

**Published:** 2025-11-08

**Authors:** Toshiyuki Terunuma, Kenta Takada, Seishin Takao, Mayu Osugi, Naoki Miyamoto, Suzuka Asano, Shunsuke Moriya, Takeji Sakae, Hideyuki Sakurai

**Affiliations:** ^1^ Institute of Medicine University of Tsukuba Ibaraki Japan; ^2^ Proton Medical Research Center University of Tsukuba Hospital Ibaraki Japan; ^3^ Graduate School of Radiological Technology Gunma Prefectural College of Health Sciences Gunma Japan; ^4^ Faculty of Engineering Hokkaido University Hokkaido Japan; ^5^ Therapy System Business, Healthcare Business Group Hitachi High‐Tech Co. Chiba Japan

**Keywords:** FPD, proton beam, secondary radiation

## Abstract

**Background:**

Secondary‐radiation background (BG) generated in kilovoltage X‐ray flat‐panel detectors (FPDs) under scanning proton beam irradiation has not been thoroughly analyzed.

**Purpose:**

This study aimed to determine and mathematically model the characteristics of secondary‐radiation BG in FPDs under scanning proton beam irradiation.

**Methods:**

Using a synchrotron‐based proton system and two FPDs mounted on a gantry tilted at 135° to the proton beam axis, we acquired images of two phantoms (block and thorax). The FPD images were captured during three conditions: only X‐ray exposure, proton irradiation, and pauses between proton irradiations. Because the FPD readout rate (30 fps) was twice the X‐ray exposure rate (15 pps), the images were further categorized into two types: those corresponding to with and without X‐ray exposure. These FPD images were analyzed to determine the characteristics of the secondary‐radiation BG. In addition, a Monte Carlo simulation was conducted to complement the performed measurements.

**Results:**

Analysis of the FPD images without X‐ray exposure revealed that the pure secondary‐radiation BG appeared as sparse spike patterns without a noticeable spatial bias across the FPD area in most images. The BG affected only 1.25% of the FPD pixels and could be modeled as an exponentially decreasing function with increasing pixel intensity. When considering typical X‐ray image brightness for the block and thorax phantoms, the impact of the BG on mean‐pixel‐intensity variation was < 1%. Monte Carlo simulations suggested that prompt photons were the primary source reaching the FPD.

**Conclusions:**

This study demonstrates that the secondary‐radiation BG characteristics observed in the X‐ray FPD images during scanning proton beam irradiation can be quantitatively modeled. The impact of the secondary‐radiation BG on mean‐pixel‐intensity of FPD images was minimal.

## 1 INTRODUCTION

Proton beam irradiation of a medium results in various forms of secondary radiation, including prompt gammas, neutrons, pair annihilation photons, bremsstrahlung, electrons, and light particles, such as deuterons, tritons, and alpha particles.^1^, ^2^ In medical applications, these secondary radiations present benefits and drawbacks. Although these secondary radiations can be applied to estimate the involved proton dose distribution, they also cause secondary‐radiation background (BG) in kilovoltage (kV) X‐ray images acquired using flat‐panel detectors (FPDs), potentially degrading image quality.

Many studies have explored the use of proton‐induced secondary radiations as beneficial signals for dose estimation in phantoms and patients. Litzenberg et al.^3^ reported a phantom study using positron emission tomography (PET) to detect 0.511‐mega‐electron‐volt (MeV) photons from electron–positron annihilation, primarily originating from ^15^O and ^11^C, which have long half‐lives of 2 and 20 min, respectively. Parodi et al.^4^ studied patients’ PET scans acquired after proton irradiation.^4^ Nishio et al.^5^ obtained online PET images of patients using a PET system mounted on a proton gantry. More recently, Ozoemelam et al.^6^ reported quasi‐prompt annihilation photons from activated ^12^N, with a half‐life of 11 ms. Regarding prompt gamma rays, Verburg et al.^7^ measured emissions at 4.44 and 6.13 MeV from proton‐induced reactions on ^12^C and ^16^O, respectively. Berthold et al.^8^ demonstrated proton range prediction using prompt gamma imaging during proton therapy. Yamaguchi et al.^9^ and Ralite et al.^10^ conducted simulations and measurements to characterize bremsstrahlung emitted by recoiled delta‐ray electrons under proton irradiation.

Besides exhibiting these beneficial applications, proton‐induced secondary radiations may produce unwanted BG signals in kV X‐ray images acquired using FPDs under proton irradiation. In addition to these secondary radiations, proton‐induced neutrons also reach the FPDs.^11^, ^12^, ^13^ These secondary radiations may degrade X‐ray FPD image quality and interfere with tumor tracking for image‐guided radiotherapy (IGRT). In the research field of therapeutic megavolt (MV) X‐ray beams, several studies have examined the BG signals from scattered MV beams or secondary radiations on kV X‐ray FPD images in terms of their magnitude, spatial distribution, and impact on cone beam computed tomography.^14^, ^15^, ^16^ However, no analyses have reported on the characteristics of secondary‐radiation BG in FPD images under scanning proton beam irradiation, leaving this phenomenon insufficiently understood.

Understanding and modeling the secondary‐radiation BG characteristics of kV X‐ray images during proton beam irradiation are increasingly important for simulating realistic X‐ray images. Such images are essential for implementing deep learning (DL) studies in IGRT, particularly for DL training. For instance, a promising tumor‐tracking concept using a patient‐specific DL model trained on individually simulated digitally reconstructed radiographs (DRRs) from computed tomography data was presented,^17^ and its feasibility was confirmed retrospectively using the clinical kV X‐ray images of 10 patients with lung cancer.^18^ However, secondary‐radiation BG modeling in training DRRs was inadequate. Given the simulation‐based, data‐driven nature of a DL method, secondary‐radiation BG must be modeled and included in the training DRRs to minimize the image quality gap between the training DRRs and actual X‐ray images under proton beam irradiation.

This study aimed to measure secondary‐radiation BG in X‐ray FPD images under scanning proton beam irradiation and to model its characteristics mathematically.

## 2 MATERIALS AND METHODS

**FIGURE 1:**
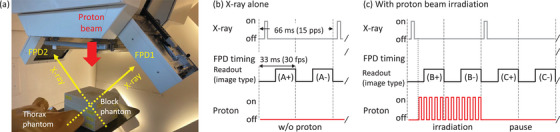
Experimental setup and timing structure. Panel (a) shows the experimental setup and equipment arrangement. Panels (b) and (c) illustrate the timing structure of X‐ray exposure, proton irradiation, and FPD image acquisition. FPD images recorded at each time point were categorized as A^+^, A^−^, B^+^, B^−^, C^+^, and C^−^, where superscripts “+” and “−” indicate the presence and absence, respectively, of X‐ray exposure.

An experiment was conducted at the Proton Beam Therapy Center, Hokkaido University Hospital. The experiment involved the use of a synchrotron‐based proton therapy machine (PROBEAT‐RT; Hitachi, Ltd. Japan) equipped with two FPDs (PaxScan 3030+; Varian Medical Systems, Inc., USA) symmetrically tilted at 135° to the proton beam axis. The target phantoms were a water‐equivalent epoxy block phantom (PH‐40; Kyoto Kagaku Co., Japan) and a custom dynamic thorax phantom (LUNGMAN + PH39; Kyoto Kagaku Co., Japan), as illustrated in Figure 1(a). Detailed parameters for X‐ray exposure and scanning proton beam irradiation are provided in Table 1.

**TABLE 1:** Experimental parameters.

Category	Parameter	Block phantom experiment	Thorax phantom experiment
Phantom	size	30 × 30 × 20 cm^3^	43 × 38 cm^2^, chest girth 94 cm
	material	epoxy	urethan, epoxy, etc.
	structure	uniform	outer: muscle, bone inner: lung, heart, diaphragm
	typical density	1.018 g/cm^3^	soft tissue: 1.06 g/cm^3^, bone: 1.31 g/cm^3^
	composition (%) or CT value	C(68.9), O(17.9), H(8.6), Ca(2.3), N(2.2), Cl(0.1)	−1000–423 HU (95% confidential interval)
Target	dummy tumor	virtually defined (no physical insert)	3‐cm‐ϕ sphere wood (–150 HU) in lung
	motion (waveform / amplitude / cycle)	static	inner structure: sin^4^/20 mm/4 s
	clinical target volume (CTV)	2.8‐cm‐ϕ sphere	3.6‐cm‐ϕ sphere
	margin ^a)^ (proximal / lateral / distal)	3/4/4 mm	2/4/4 mm
	maximum depth	12.5 cm	7.5 cm
Proton	energy ^b)^	108–152 MeV	103–139 MeV
	total dose (max. rate = 10 MU/s (net))	1.0 GyE (RBE 1.1)	3.5 GyE (RBE 1.1)
	total spots ^c)^ and layers	726 spots, 16 layers	5203 spots, 13 layers
	spot interval (spot size ^d)^ = 4 mm (1σ))	5.3 mm	3 mm
X‐ray	tube parameter and exposure rate	low: 70 kV, 10 mA, 3 ms, 15 pps high: 125 kV, 80 mA, 3 ms, 15 pps	80 kV, 40 mA, 3 ms, 15 pps
	field at isocenter	10 × 10 cm^2^	21 × 21 cm^2^
FPD	position	60 cm from isocenter, 135° to proton beam axis	←
	size and pixel number	298 × 298 mm, 768 × 768 (2 × 2 binned)	←
	frame rate	30 fps	←

^a)^ Beam‐specific margins, accounting for internal motion and setup uncertainty.

^b)^ Proton energy was optimized to ensure adequate target coverage.

^c)^ The number of spots was automatically calculated based on the dose limit per spot (0.05 MU).

^d)^ Measurements were taken at the isocenter in air. Abbreviations: Hounsfield unit (HU), gray equivalent (GyE), relative biological effectiveness (RBE), monitor unit (MU), flat‐panel detector (FPD), pulse per second (pps), frame per second (fps).

The experiments were performed under two scenarios for each phantom: FPD imaging under kV X‐ray alone without proton irradiation (Figure 1(b)) and FPD imaging under kV X‐ray with proton beam irradiation (Figure 1(c)). In addition, to minimize lag effects caused by residual signals in the FPD images,^19^ a baseline experiment was performed using the lowest available X‐ray tube settings, as shown in Table 1. Because the X‐ray exposure rate was 15 pulse‐per‐second (pps) and the FPD readout rate was 30 frame‐per‐second (fps), the recorded FPD images were categorized into two types, labeled A^+^ and A^−^ in Figure 1(b). Superscripts “+” and “−” indicate the presence and absence, respectively, of X‐ray exposure. FPD imaging under proton irradiation is shown in Figure 1(c). It is important to note that the proton therapy system did not simultaneously irradiate X‐rays and protons because of a regulation prohibiting the simultaneous use of the two radiation sources. Therefore, images B^+^ and B^−^ were captured during proton beam irradiation; however, for image B^+^, proton beam irradiation was halted during the pulsed X‐ray exposure and resumed after the X‐ray exposure owing to the abovementioned regulation. In other words, image B+ contained the X‐ray image and secondary‐radiation BG produced during proton irradiation, lasting ∼20 ms, though the vendor did not disclose the exact value. Image B^−^ contained only the secondary‐radiation BG generated during proton irradiation (∼33 ms). Images C^+^ and C^−^ were captured during pauses between proton irradiations, which occurred during energy layer transitions for volumetric target irradiation and due to the inherent characteristics of the proton synchrotron, which does not continuously extract proton beams. Additional information regarding the timing structure is available in previous reports.^20^, ^21^ Therefore, the predicted primary BG sources in the B images acquired during proton beam irradiation were neutrons and “prompt photons,” including not only prompt gamma rays but also bremsstrahlung and annihilation photons. In contrast, those in the C images acquired at the proton irradiation intervals were “delayed photons” originating from activated nuclei under proton beam irradiation. Naturally, all acquired images contained thermal noise from the FPD itself.

Based on the experimental parameters, we simulated proton beam interactions with the block phantom and tracked the emission of secondary particles using the PHITS Monte Carlo package (Version 3.35).^22^ The package comprises the EGS5 code^23^ for electrons and photons and the JENDL‐4 library^24^ for neutrons and protons. The bremsstrahlung emitted from recoil electrons with energies above 10 keV were evaluated using the delta‐ray section of PHITS. However, the generation and decay of radioisotopes under proton irradiation were not considered. Calculations were accelerated using the supercomputer *Miyabi* (see Acknowledgments). Figure 6(a) in Results section illustrates the simulation setup. A total of 2.5 × 10^7^ proton histories were simulated to analyze the spatial distributions and energy spectra of prompt photons and neutrons at the FPD position. To assess spatial BG variation, the FPD was divided into three sections (#3, #4, and #5 in Figure 6(a)).

## 3 RESULTS

**FIGURE 2:**
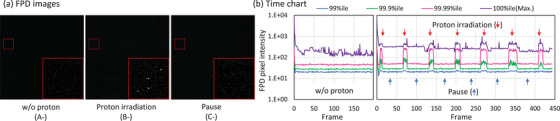
FPD images and time chart. Panel (a) presents example FPD images; the small red regions containing spike signals are enlarged at the lower‐right of each image. Panel (b) presents a time chart showing the variations in Nth percentile pixel intensity across sequential FPD images. Image types A, B, and C correspond to those defined in Figure 1.

**FIGURE 3:**
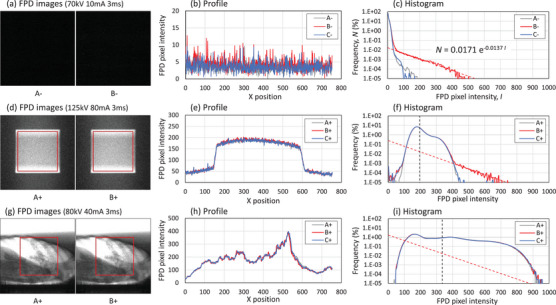
FPD images, central x‐line profiles, and histograms. Panels (a)–(c) show pure background data without X‐ray exposure; (d)–(f) and (g)–(i) present X‐ray image data for the block and thorax phantom, respectively. Histograms (f) and (i) were calculated from the red region of interest only. Image types A, B, and C correspond to those in Figure 1. The red dotted line and equation in (c) indicate exponential fitting in the 100–300 pixel intensity range. In (f) and (i), the red fitting line from panel (c) was shifted horizontally by the mean pixel intensity (black dotted line) and overlaid.

To quantify the pure secondary‐radiation BG independently of the X‐ray image contributions, we analyzed the FPD images captured without X‐ray exposure: image types A^−^, B^−^, and C^−^. As shown in Figure 2(a), the sparse spike‐pattern BG resulting from the secondary radiations appeared in the FPD image during proton irradiation. A time chart of the Nth percentile pixel intensity of the FPD images indicated a rapid decrease in pixel intensity (Figure 2(b)). The histograms obtained during the X‐ray‐only period (A^−^) and proton irradiation pause (C^−^) exhibited no notable difference in pixel value distribution. Meanwhile, secondary‐radiation BG was observed in the B^−^ FPD image acquired during proton irradiation. To quantify the BG, an exponential fit was applied to the relevant portion of the histogram presented in Figure 3(c). The percentage of secondary‐radiation BG (*N*) was modeled as a function of pixel intensity (*I*) as follows:

(1) $$N=k\;e^{-\lambda I}$$

Herein, the percentage amplitude k was approximately 0.017% and coefficient λ was approximately 0.014. These values for the two FPDs were almost the same. By integrating Equation 1 over the pixel intensity range from 0 to 1000, the number of pixels affected by the secondary‐radiation BG was estimated to be 1.25% of the total FPD pixels.

Table 2 shows the variations in mean pixel intensity for each phantom and experimental setting. During proton irradiation, the mean pixel intensity of dark FPD images (i.e., lowest X‐ray exposure) increased by 8.6% relative to baseline. For typical clinical X‐ray image brightness, the resulting change in mean pixel intensity was < 1%.

**TABLE 2:** Variation of FPD pixel intensity.

Experiment	Block phantom									Thorax phantom		
X‐ray tube parameter	Low (70 kV, 10 mA, 3 ms)						High (125 kV, 80 mA, 3 ms)			80 kV, 40 mA, 3 ms		
X‐ray exposure	Off			On			On			On		
FPD image timing	w/o P.	P. irrad.	pause	w/o P.	P. irrad.	pause	w/o P.	P. irrad.	pause	w/o P.	P. irrad.	pause
FPD image type	A−	B−	C−	A+	B+	C+	A+	B+	C+	A+	B+	C+
Mean pixel intensity	5.0	5.5	5.0	5.5	5.9	5.6	195.9	196.9	195.8	335.7	334.6	336.9
Variation from w/o P.	−	0.6	0.1	−	0.5	0.1	−	1.0	−0.1	−	−1.1	1.2
Relative variation %	−	12%	1.4%	−	8.6%	1.9%	−	0.5%	−0.1%	−	−0.3%	0.3%

Image types A, B, and C correspond to those defined in Figure 1. Abbreviations: proton (P.), irradiation (irrad.).

**FIGURE 4:**
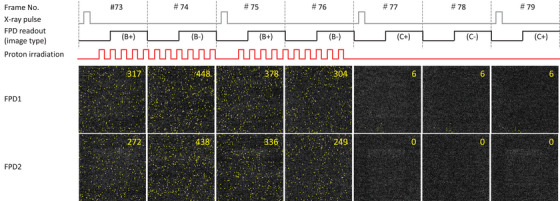
Examples of FPD images with the time chart. Frame numbers with the symbol “#” correspond to the time chart in Figure 2. The window width of the FPD images was adjusted to 25 for visualization. The large yellow dots mark pixels with intensities exceeding 100, and the numbers shown in each image indicate the total dot count.

**FIGURE 5:**
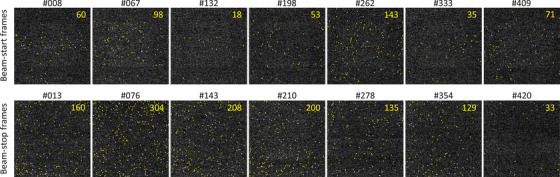
Examples of FPD images at the beam‐start and ‐stop frames. (top) FPD1 images at the beam‐start frames. (bottom) FPD1 images at the beam‐stop frames. Frame numbers with the symbol “#” correspond to the time chart in Figure 2. The window width of the FPD images was adjusted to 25 for visualization. The large yellow dots mark pixels with intensities exceeding 100, and the numbers shown in each image indicate the total dot count.

An example of the sequential FPD images, including the noisiest one, is shown in Figure 4 with its time chart. Pixels exceeding the threshold brightness (100) were marked with enlarged yellow dots. The dot count during proton irradiation was rapidly decreased during the pause phases. In the FPD1 images acquired during the pause phases, only one pixel was likely affected by secondary‐radiation BG, assuming the other five fixed pixels had large offsets due to their inherent pixel‐specific characteristics. The spatial secondary‐radiation BG distribution during proton irradiation was not noticeably biased (Figure 4); however, a thorough analysis of the frame images corresponding to the beginning and end of scanning proton beam irradiation (Figure 5) revealed that the BG distributions in a few images exhibited large spatial biases. This was attributed to the signal readout mechanism of the FPDs (the details are presented in Discussion and Supplementary Figure).

**FIGURE 6:**
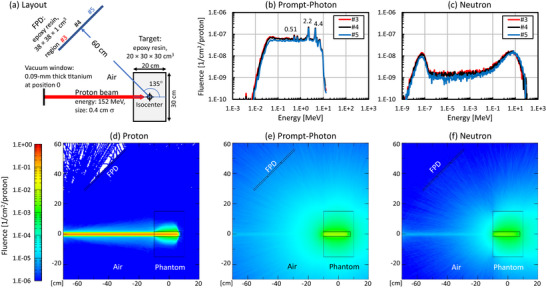
Results of PHITS calculations. (a) Simulation layout, (b) prompt‐photon energy spectrum at the FPD position, (c) neutron energy spectrum at the FPD position, (d) proton distribution, (e) prompt‐photon distribution, and (f) neutron distribution. The FPD region was divided into three sections—near, middle, and far—relative to the beam axis and labeled as regions #3, #4, and #5, respectively.

The PHITS simulation results are shown in Figure 6. The peak energies of 4.4, 2.2, and 0.51 MeV in Figure 6(b) correspond to prompt photons from the de‐excitation of the ^12^C(p,p’)^12^C^*^ reaction, the ^1^H(n,γ)^2^H reaction, and annihilation photons, respectively. The numbers of prompt photons per proton (1/cm^2^/proton) at the three FPD regions (#3, #4, and #5) were 5.09 × 10^−6^ (105%), 4.83 × 10^−6^ (100%), and 4.10 × 10^−6^ (85%), respectively, with percentages in parentheses indicating values relative to the center region #4. The numbers of neutrons per proton (1/cm^2^/proton) at these regions were 1.34 × 10^−6^ (106%), 1.26 × 10^−6^ (100%), and 1.06 × 10^−6^ (84%), respectively.

## 4 DISCUSSION

To the best of our knowledge, this research presents the first analysis of secondary‐radiation BG based on the frame‐by‐frame measurements of kV X‐ray FPD images during scanning proton beam irradiation. Herein, we address key aspects of the BG characterization: the estimation of absolute quantities, spatial distributions, and mathematical modeling.

The time‐dependent behavior of secondary‐radiation BG was analyzed using the sequential FPD images (Figures 2, 3, 4). The BG signals were observed as sparse spike patterns in the FPD images during proton irradiation; meanwhile, they rapidly diminished during the pause phases. Approximately 1.25% of the FPD pixels were affected during proton irradiation. For typical X‐ray image brightness, the variation in the mean pixel intensity was < 1% (Table 2). As X‐ray image brightness and secondary‐radiation BG are independent, it is valid to simulate the BG histogram during proton irradiation as an additive component to the X‐ray‐only image, as shown in the histogram in Figure 3(f).

The spatial distribution of secondary‐radiation BG on the FPD was then examined. Although PHITS simulations indicated a bias of 15% for prompt photons and 16% for neutrons across the FPD regions, no noticeable spatial imbalance was observed in the measured BG distribution (Figure 4). In our proton therapy system, the FPDs were positioned at 135° relative to the proton beam axis from the isocenter (Figure 1(a)). This arrangement helps mitigate the influence of forward‐directed secondary radiation on FPD images. Prompt gamma rays, bremsstrahlung, and neutrons have anisotropic forward‐biased angular distributions; whereas, their emissions in the backward direction exhibit substantially reduced angular dependence.^9^, ^25^, ^26^ Despite these general trends, several beam‐start and beam‐stop frames exhibited pronounced spatial imbalance in the BG distribution (Figure 5). This anomaly arose from the interplay between the proton beam timing and the FPD readout. Large‐area FPDs are typically constructed by tiling smaller units, with pixel charges read sequentially from the periphery toward the center. As the FPD readout timing is independent of the synchrotron operation cycle and spot irradiation completion, a slight delay in beam initiation or an abrupt termination during the FPD readout can result in the spatially biased distribution of the BG. A detailed explanation of this mechanism is provided in Supplementary Figure. Nonetheless, the majority of FPD images did not exhibit noticeable BG spatial imbalance. Considering these factors, we concluded that the spatial distribution of secondary‐radiation BG in most FPD images can be modeled as a random process without spatial bias.

**FIGURE 7:**
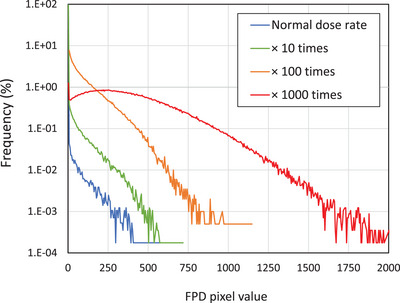
Simple simulation of the pile‐up effect in response to beam intensity.

We then validate the mathematical modeling of secondary‐radiation BG. Because high‐energy photons and neutrons fall outside the intended operational range of the FPD (designed for X‐ray imaging at tube voltages of 40–150 kVp), there are few direct references to these interactions. For the thin thallium‐doped cesium iodide (CsI(Tl)) scintillator used in the FPD, the mass attenuation coefficient of photon decreases with increasing photon energy, reaching a plateau around 5 MeV.^27^ The cross sections of neutrons generally decrease with increasing energy, following the 1/velocity law, except for resonance. Takada et al., who focused on a neutron‐and‐photon mixed radiation field for boron neutron capture therapy, suggested similar trends.^28^ Using thick lithium fluoride and a thin silicon‐diode detector, they reported that counts of a multichannel analyzer (MCA) decreased with increasing pulse heights in calibration using ^60^Co photon sources. This trend resembles that shown in Equation 1. However, they also noted distortions in the response curve at high dose rates due to pile‐up phenomena. To simulate this pile‐up effect, we calculated BG accumulation, which was randomly distributed according to Equation 1 across a 768 × 768‐pixel matrix, representing a virtual FPD. As shown in Figure 7, the BG curve followed Equation 1 up to dose rates 100 times higher than the normal; however, the pile‐up effect became apparent at rates exceeding 1000 times the normal dose rate.

To compare the secondary‐radiation BG generated under MV therapeutic photon beam irradiation, the studies by Iramina et al.^15^ and Nguyen et al.^16^ were referenced. Iramina et al.^15^ showed that, in their figure 3(a), the mean FPD pixel intensity for kV X‐ray‐only imaging at 15 fps was approximately 3000 for a 3 × 3 cm^2^ field size, increasing to 3300 under 6 MV photon irradiation with a flattening filter (FF). This corresponds to an approximately 1.7% increase when applying a simple correction for the FPD frame rate and the beam field size under our proton irradiation conditions—30 fps and a 10 mm (σ) beam size at the Bragg peak (BP) in the targets (Table S1). Nguyen et al.^16^ demonstrated that, in an image profile (their figure 5(c)), a pixel value for kV X‐ray‐only rose from approximately 2100 to 4200 under 6 MV photon irradiation using the FF‐free mode for a 6 × 6–cm^2^ field size at 7 fps. This corresponds to an approximately 2% increase after applying the corrections (Table S2). In contrast, the secondary‐radiation BG from scanning proton beam irradiation resulted in < 1% variation in the mean pixel intensity (Table 2).

This study has several limitations. The thorax phantom experiment was originally designed to assess marker‐less tumor tracking, rather than evaluate the BG signals. During kV imaging, the respiratory motion of the internal lung structures introduced a maximum interframe displacement of 0.6 mm. This minor motion influenced the pixel intensity in the FPD images, resulting in an inversion phenomenon, wherein the mean pixel intensity during proton beam irradiation was lower than that during X‐ray imaging alone (Table 2). For exact validation, the BG experiment should be conducted without motion. However, the current findings suggest that motion‐induced brightness variations have a greater impact on X‐ray FPD images than secondary‐radiation BGs.

Although secondary‐radiation BG affected < 1% to the mean pixel intensity of the FPD at regular treatment dose rates, it can become notable if ultrahigh‐dose‐rate irradiation (FLASH) is used in the future.^29^, ^30^ Research on FLASH therapy indicates that a dose rate exceeding 40 Gy/s is generally required to achieve the FLASH effect, which enhances tumor control while minimizing damage to surrounding healthy tissues. Conventional proton therapy is typically performed at dose rates of 0.01–0.1 Gy/s, meaning that the FLASH dose rate is 100–1000 times higher. The pile‐up simulation of the BG at such high dose rates, as shown in Figure 7, suggests that Equation 1 may not be applicable when dose rates are 1000 times higher. Future work should evaluate the secondary‐radiation BG behavior at a high dose rate.

Our synchrotron‐based proton system has a lower beam intensity than other cyclotron‐based systems, resulting in lower secondary‐radiation BG. In addition, when the FPD is located near the beam forward direction (e.g., at a 45° angle on a gantry) or with a gantry angled toward a ceiling‐mounted FPD, the BG levels may increase. However, under standard beam intensity, the BG characteristics modeled by Equation 1 can be applied with parameter adjustment to most proton therapy systems owing to their similar FPD design.

We recognize the uncertainty in comparing secondary‐radiation BG between MV photon beams and proton beams due to notable differences in equipment characteristics and irradiation methods. Although we corrected the BG levels using the FPD frame rate and MV beam field size (Supplementary Tables), the latter did not reflect an actual mean field size during intensity‐modulated radiotherapy (IMRT) for patients. While the minimum field size during IMRT may be comparable to the typical scanning proton beam size at the BP position, the maximum field size in IMRT is substantially larger. Therefore, our field‐size correction might be an overcorrection of the BG level for IMRT.

In PHITS simulations, the generation and decay of radioisotopes during and after proton irradiation were not considered. Consequently, the photon yield may have been slightly underestimated. The difference in the total number of photons and neutrons reaching the FPD was minimal (< five times), rendering it difficult to identify which component was the primary source of the spike pattern. A detailed simulation of particle‐to‐light conversion in the thin CsI(Tl) scintillator layer and subsequent light‐to‐signal conversion in the photodiodes would be necessary to identify the contribution.

This BG modeling study was performed to calculate the realistic training DRRs for DL‐based real‐time marker‐less tumor tracking. However, the effectiveness of secondary‐radiation BG modeling in terms of tracking accuracy is beyond the scope of this report and will be addressed in future research.

## 5 CONCLUSIONS

This research provides the first quantitative assessment of secondary‐radiation BG in kV X‐ray FPD images during scanning proton beam irradiation. The secondary‐radiation BG appeared as sparse spike patterns without noticeable spatial bias in most of the FPD images. It appeared on approximately 1.25% of the FPD pixels and affected < 1% of the brightness of typical X‐ray images. Finally, the characteristics of secondary‐radiation BG could be mathematically modeled as an exponentially decreasing response to the pixel intensity.

## CONFLICTS OF INTEREST STATEMENT

**M.O**. and **S.A**. are employed by Hitachi High‐Tech Co. **T.S**. has been supported by funding from Hitachi High‐Tech Co. since 2024. **T.T**. had a software agreement on a deep learning–based tumor‐tracking method with Hitachi Ltd. from 2022 to 2024. **H.S**. has received honoraria from Hitachi High‐tech Co. All other authors declare no competing interest.

## Supporting information



Supporting Tables

Supporting Figure
