# Supplementary material for: Characterization of secondary‐radiation background in X‐ray flat‐panel detectors during scanning proton beam irradiation
Source: Med Phys. 2025 Nov 8;52(11):e70121. doi: 10.1002/mp.70121 (PMC12629891; doi:10.1002/mp.70121)
Supplement: Supplementary file 1 — Supporting Tables [file MP-52-0-s002.pdf]

Supplementary Table 1 (S1)

	**Reference 16 (Iramina et al.)**	**Our block phantom**
Phantom	40-cm-ϕ cylindrical phantom (plastic)	30 × 30 × 20 cm^3 epoxy phantom
Beam type	6 MV photon beam (with Flattening Filter)	Scanning proton pencil beam
Dose rate	10 MU/s (600 MU/min)	10 MU/s (net)
Field (beam) size	3 × 3 cm^2 (9 cm^2)	σ ≈ 0.1 cm at Bragg peak (3.14 cm^2)
KV X-ray parameters	125 kV, 60 mA, 20 ms pulse	125 kV, 80 mA, 3 ms pulse
Frame rate	15 fps (66 ms/frame)	30 fps (33 ms/frame)
FPD pixel value	kV only: 3000<br>kV + MV: 3300<br>(Their Fig. 3(a))	kV only: 195.9<br>kV + proton: 196.9<br>(Table 2)
Raw variation	**+ 10.0%**	**+ 0.5%** (Table 2)
Correction factors	frame rate: 15 / 30 = 0.5<br>field size: 3.14 / 9 ≈ 0.35	
Corrected variation	**10.0%** × **0.5** × **0.35** → **+ 1.75%**	**+ 0.5%** (no correction)

Supplementary Table 2 (S2)

	**Reference 17 (Nguyen et al.)**	**Our thorax phantom**
Phantom	Torso Phantom (Kyoto Kagaku. Co. Ltd. Japan)	Thorax phantom (Kyoto Kagaku. Co. Ltd. Japan)
Beam type	6 MV beam (Flattening-Filter Free: FFF)	Scanning proton pencil beam
Dose rate	23 MU/s (1400 MU/min)	10 MU/s (net)
Field (beam) size	6 × 6 cm^2 (36 cm^2)	σ ≈ 0.1 cm at Bragg peak (3.14 cm^2)
KV X-ray parameters	120 kV, 0.3 mAs	80 kV, 40 mA, 3 ms pulse = 0.03 mAs
Frame rate	7 fps (143 ms/frame)	30 fps (33 ms/frame)
FPD pixel value	kV only: 4200<br>kV + MV: 2100<br>(Their Fig. 5(c), at position 400)	kV only: 335.7<br>kV + proton: 334.6 ~ 336.9<br>(Table 2) Slight phantom motion affected the measured values.
Raw variation	**+ 100%**	**± 0.3%** (Table 2)
Correction factors	frame rate: 7 / 30 ≈ 0.23<br>field size: 3.14 / 36 ≈ 0.087	
Corrected variation	**100%** × **0.23** × **0.087** → **+ 2.0%**	**± 0.3%** (no correction)
