# Supplementary figures and images for: Characterization of secondary‐radiation background in X‐ray flat‐panel detectors during scanning proton beam irradiation
Source: Med Phys. 2025 Nov 8;52(11):e70121. doi: 10.1002/mp.70121 (PMC12629891; doi:10.1002/mp.70121)

Supplementary Figure

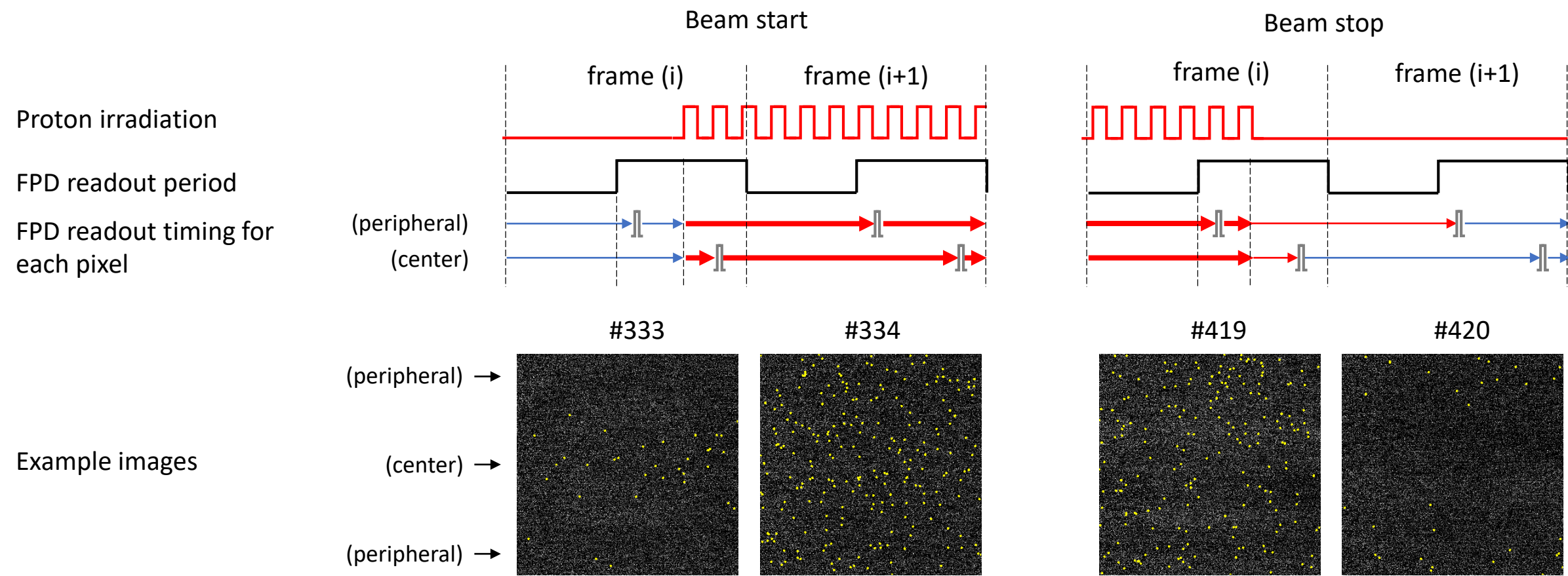

Supplement: Supplementary file 2 — Supporting Figure [file MP-52-0-s001.pdf]
